# High-Density, Actively Multiplexed μECoG Array on Reinforced Silicone Substrate

**DOI:** 10.3389/fnano.2022.837328

**Published:** 2022-02-24

**Authors:** Iakov Rachinskiy, Liane Wong, Chia-Han Chiang, Charles Wang, Michael Trumpis, John I. Ogren, Zhe Hu, Bryan McLaughlin, Jonathan Viventi

**Affiliations:** 1 Department of Biomedical Engineering, Duke University, Durham, NC, United States,; 2 Micro-Leads Inc., Somerville, MA, United States,; 3 Department of Neurobiology, Duke University School of Medicine, Durham, NC, United States,; 4 Duke Institute of Brain Sciences, Duke University School of Medicine, Durham, NC, United States,; 5 Department of Neurosurgery, Duke University School of Medicine, Durham, NC, United States

**Keywords:** μECoG, neural interface, actively multiplexed, implanted device, flexible substrate

## Abstract

Simultaneous interrogation of electrical signals from wide areas of the brain is vital for neuroscience research and can aid in understanding the mechanisms of brain function and treatments for neurological disorders. There emerges a demand for development of devices with highly conformal interfaces that can span large cortical regions, have sufficient spatial resolution, and chronic recording capability while keeping a small implantation footprint. In this work, we have designed 61 channel and 48 channel high-density, cortical, micro-electrocorticographic electrode arrays with 400 μm pitch on an ultra-soft but durable substrate. We have also developed a custom multiplexing integrated circuit (IC), methods for packaging the IC in a water-tight liquid crystal polymer casing, and a micro-bonding method for attaching the electronics package to the electrode array. With the integrated multiplexer, the number of external wire connections can be reduced to 16 wires, thereby diminishing the invasive footprint of the device. Both the electrode array and IC were tested *in vivo* in a rat model to demonstrate the ability to sense finely-localized electrophysiological signals.

## INTRODUCTION

Brain function is composed of coordinated activity and interaction between populations of neurons distributed across large areas of the brain. Electrical signals produced by this neural activity are studied to answer questions in neuroscience, diagnose neurological disorders, identify pathological neural tissue in the clinical application, and interpret brain activity for use in brain computer interfaces (BCIs) (Schroeder & Chestek, 2016; Anyanwu & Motamedi, 2018; Milekovic et al., 2018; Rabbani et al., 2019). Acquiring electrical signals from large and distributed brain areas with both high spatial and temporal resolution is vital for these applications and requires novel neural interface technology that can provide high-resolution sampling and improved longevity of the implanted devices (Slutzky et al., 2010; Chang, 2015).

Micro-electrocorticography (μECoG) is a technique that uses arrays of sub-millimeter sized electrodes in a two-dimensional grid to sense electrical brain activity from the surface of the brain (Shokoueinejad et al., 2019). Recording from the surface is less damaging to brain tissue and therefore allows for development of devices with larger spatial coverage and greater numbers of recording sites than current intra-cortical recording methodologies can offer (Wang et al., 2009; Shokoueinejad et al., 2019). However, scaling μECoG devices to cover large areas while preserving small electrode pitch requires large numbers of electrode channels, presenting wiring challenges if active multiplexing is not utilized.

For large spatial coverage, devices must conform to the curved surface of the brain to access cortical regions that may have complex and uneven surface geometries. Conformity of the device ensures that sufficient electrode proximity to the firing neural tissue is maintained upon implantation to acquire strong electrical signals (Buzsáki et al., 2012). Fabricating such conformal devices necessitates developing soft, flexible substrates that can mimic the curvature of the cortex while also providing sufficient mechanical strength to protect the embedded electrical features of the device from bending, stretching, or repetitive stress from pulsatile brain movement (Chen et al., 2017). Additionally, matching mechanical properties between the neural interface and the brain tissue can reduce inflammation, tissue damage and decrease the foreign body response due to the implant (Araki et al., 2020). This, in turn, can improve signal quality and longevity of the device.

Scaling the number of contacts to achieve high spatial resolution recordings over large areas demands solving the wiring problem: how to connect large numbers of electrodes to an external recording system outside of the skull while satisfying anatomic size constraints and mitigating infection risks caused by percutaneous tethering. The craniotomy limits the area through which wires can emerge from within the skull, putting a hard threshold on the number of passively wired electrodes that can be implemented on a device. Emerging wires are the prime cause of infection during and after electrode implantation, as they allow access across the blood-brain barrier (Kourbeti et al., 2015). Due to these constraints, novel methods must be implemented to increase the number of contacts on μECoG devices while limiting the number of emerging wires used to connect the electrodes to the external recording system (Viventi et al., 2011; Jun et al., 2017; Tsai et al., 2018; Chiang et al., 2020).

Therefore, there is a demand for highly scalable devices that span large cortical regions and have sufficient spatial resolution. To tackle these scalability demands, we have developed methods for fabricating an actively multiplexed electrode array on a soft but robust substrate material to capture fine spatio-temporal information from the cortical surface. The novel substrate is fabricated from reinforced silicone that has the flexible and stretchable qualities of silicone but possesses improved strain and tear resistance due to reinforcement through embedded microfibers within the material. Laser-machined metal traces made from ultra-thin platinum iridium (Pt-Ir) foil provide high-density routing and a large number of micro-electrode sites. A customized application-specific integrated circuit (ASIC) embedded directly on the electrode substrate multiplexes the electrode sites to reduce the number of emerging wires for communication with the external recording system and safely operates from AC power. We tested the capabilities of the substrate and the customized ASIC in a rat model to demonstrate the ability to record neural signals at high spatial density from a reduced number of wires with the novel electrode and recording hardware.

## METHODS

### Reinforced Silicone Substrate

**FIGURE 1 |:**
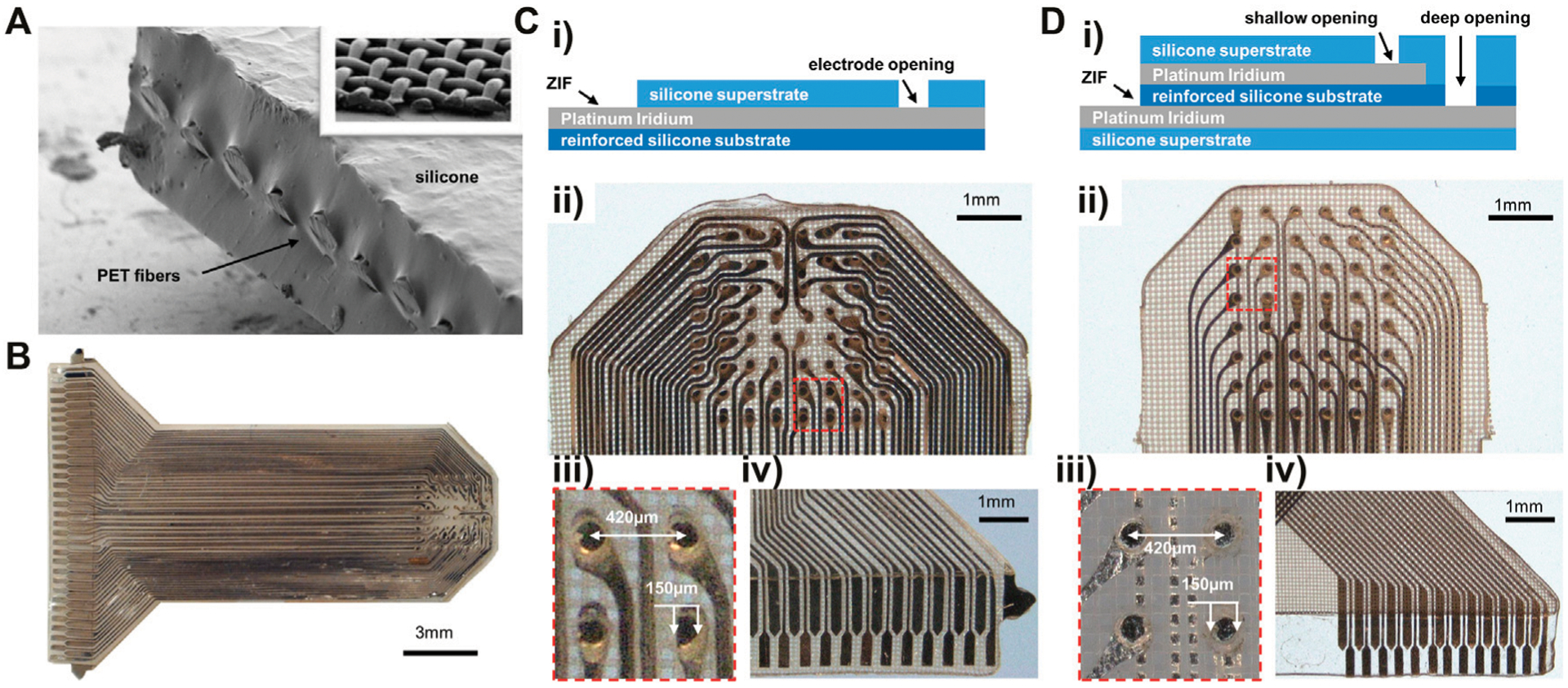
Materials and passive electrode array fabrication. **(A)** SEM of reinforced silicone substrate cross section, with inset showing the interwoven PET micro-threads in top right. **(B)** 61-channel monolayer reinforced silicone electrode array. **(Ci)** Stackup for monolayer electrode array fabrication. **(Cii)** Monolayer reinforced silicone uECoG array contacts. **(Ciii)** Close-up of four electrode sites in Cii. **(Civ)** Exposed pads for ZIF connector attachment. The horizontal line shows where the superstrate ends to keep the ZIF pads exposed for contact. **(Di)** Stackup for two-metal layer electrode array fabrication. **(Dii)** Two-layer reinforced silicone electrode array with electrodes sitting at two depths within substrate. **(Diii)** Close-up of four electrodes from Dii at two depths: darker metal on the left in layer 1, fainter electrodes on right in layer two behind a layer of meshed silicone. **(Div)** ZIF connector portion of two-layer electrode with overlapping traces in top corner patterned on separate layers.

We have developed a reinforced silicone substrate that benefits from the softness and flexibility of ultra-thin silicone, but with improved mechanical properties. To do this, we embedded woven 35 μm (PET) micro-thread fabric within a 150 μm thick silicone substrate (Figure 1A). We used implant-grade component materials (Nusil MED-4174) to ensure safety during implantation of the substrate within the body. Separately, we have successfully completed ISO-10993 biocompatibility testing for human implantation (<30 days) on electrodes comprised of the same reinforced silicone substrate (NAMSA and Toxikon laboratories).

### Electrode Fabrication

The micro-mesh reinforced silicone substrate (150 μm), ultra-thin Pt-Ir conductors (18 μm), and silicone superstrate (100 μm) were precision patterned using high repetition laser ablation. The laser patterning precision is demonstrated in Figure 1B, where platinum-iridium foil is patterned into intricate traces and electrodes on a silicone substrate. The insulating superstrate silicone layer was then laminated to create a fused, biocompatible device that is thin, flexible, and resistant to tearing and delamination. A monolayer version of the electrode array was fabricated on a single metal layer with traces and electrodes all patterned on one metal plane. Electrode pads were exposed by laser-opening a circular aperture in the superstrate silicone cover (Figure 1Ci). Our electrode array design consisted of an 8 × 8 electrode grid with 61 functional sites that spanned a ~3 × 3 mm recording area (Figure 1Cii). Electrode sites had 150 μm diameter pads and 420 μm pitch, providing high-density, sub-millimeter scale sampling (Figure 1Ciii). We also designed a second version of the electrode array that used two metal layers for traces and electrode sites to demonstrate ability to fabricate devices with multiple metal layers (Figure 1Di). Two-layer devices had similar pitch and pad sizes to the monolayer design and 48 overall contacts in a 6 × 8 grid (Figure 1Dii). A zero-insertion force (ZIF) connector on the end of the electrode array was fabricated all on the same layer by leaving the metal connector pads uncovered by the superstrate silicone (Figures 1Civ,Div).

### ASIC Design

**FIGURE 2 |:**
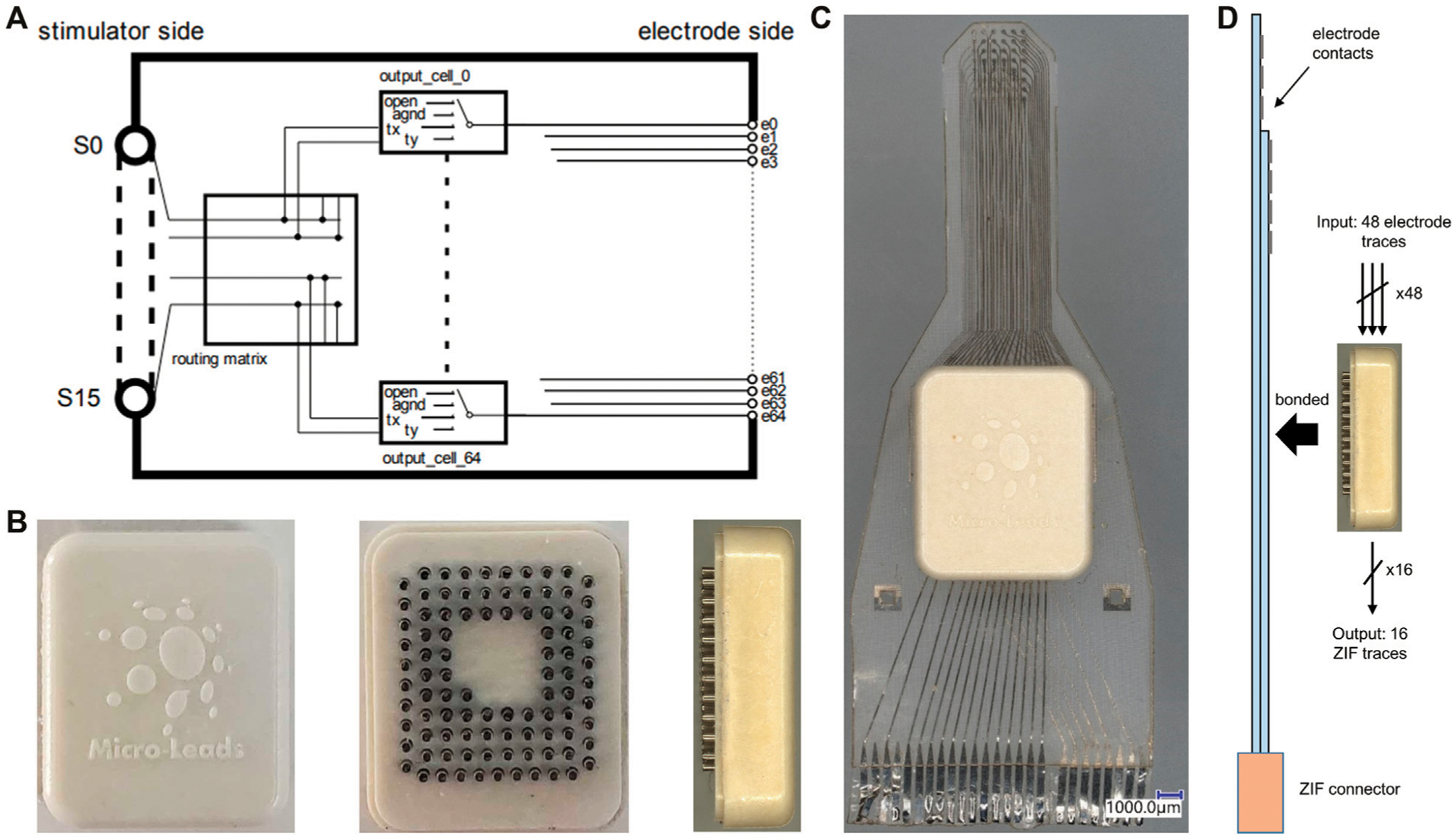
1664 ASIC and active electrode array fabrication. **(A)** Schematic of ML1664 ASIC. 64 multiplexers connect each electrode line to several output S lines. **(B)** The ASIC inside LCP packaging in top, bottom and side view from left to right. Pin-grid feedthroughs are used for thermal bonding to the electrode array substrate. **(C)** Active electrode array with embedded multiplexing ASIC within an LCP electronics package. The electronics package contained a pin-grid array bonded to the electrode array and underfilled. The active electrode used two metal layers for wiring, evident from the different shade of traces on left and right side of ZIF connector. **(D)** Diagram of pin-grid attachment to the active-electrode array using thermal bonding. 48 electrode contacts wire to inputs of 8 × 6:1 MUXs on the 1664 ASIC and 16 outputs connect *via* ZIF to recording system, thereby reducing output wire count.

We designed and fabricated a low power, high-density, analog multiplexer array (ML1664 ASIC). The ASIC contained 16 inputs with bi-directional switches at each of 64 electrode nodes. There was redundancy in the switches to enable multi-polar stimulation paradigms (Figure 2A). The input/output channels were designed for both sending stimulation to the neural tissue and sensing neural activity. The ASIC allowed for the limited number of stimulation channels on a neural stimulator to be shared across a large group of electrodes. Each electrode could be programmed to be open, grounded or connected to one of two input channels (tx, ty in Figure 2A). Thus, the ML1664 ASIC reduced the number of leads required between a pulse generator or recording system and an electrode array. At any instant, a maximum number of 16 output channels could be stimulated or sampled. However, the multiplexer allowed for rapid switching to a new configuration, thus enabling 64 electrodes to be stimulated or sampled “near simultaneously”. Stimulation currents or neural recording signals could pass through the multiplexer cells. An external algorithm could automatically connect the output and input channels as per user-defined stimulation/sensing configurations to allow for custom electrode patterns. The ASIC was fabricated using the XFAB 350 nm 50 V process. Solder balls were jetted onto a transition layer between the ASIC and the feedthrough array which enables flip-chip bonding. The AC-powered ASIC was physically small and protected from the environment using LCP-based near-hermetic packaging (7.800 × 9.375 × 2.350 mm) (Figure 2B). The pin-grid feedthrough array on the packaged ASIC could be thermally bonded to an electrode array (Figure 2D) and enabled stimulation and recording on up to 64 electrode sites.

### Active-Electrode Fabrication

Implanted electrode arrays presently require one wire for each electrode, preventing substantial scaling for high-resolution arrays. We developed an active electrode array with embedded multiplexing electronics to provide recording and stimulating on up to 64 electrodes using only 16 external wires (Figure 2C). For the active electrode array, a platinum-iridium footprint was patterned on the silicone substrate for connection to the active electronics. A pin-grid array was fabricated from injection-molded liquid crystal polymer (LCP) with platinum-iridium pins (Figure 2B). An ASIC was fabricated with a transition layer to enable flip-chip bonding of the ASIC onto the pin-grid array. After flip-chip bonding the ASIC to the pin-grid array, an underfill material was applied under the ASIC to prevent moisture ingress under the chip. An LCP lid was used to seal the package. The pins of the pin-grid array were then bonded to the high-density μECoG array using a thermal bonding process (Figure 2C). Finally, the air gap between the package and the silicone substrate was underfilled with a silicone elastomer (NUSIL MED-4211). Two metal-layers were used for the active device. Size constraints and manufacturing limitations required the design to utilize 48 channels instead of the maximum potential of 64. The 48 electrode sites were multiplexed, and 16 output wires were connected to the electrode array through a 26 pin ZIF to a recording system to provide power, clock, digital controls, reference, and analog output channels (Figure 2D).

### Recording Setup

**FIGURE 3 |:**
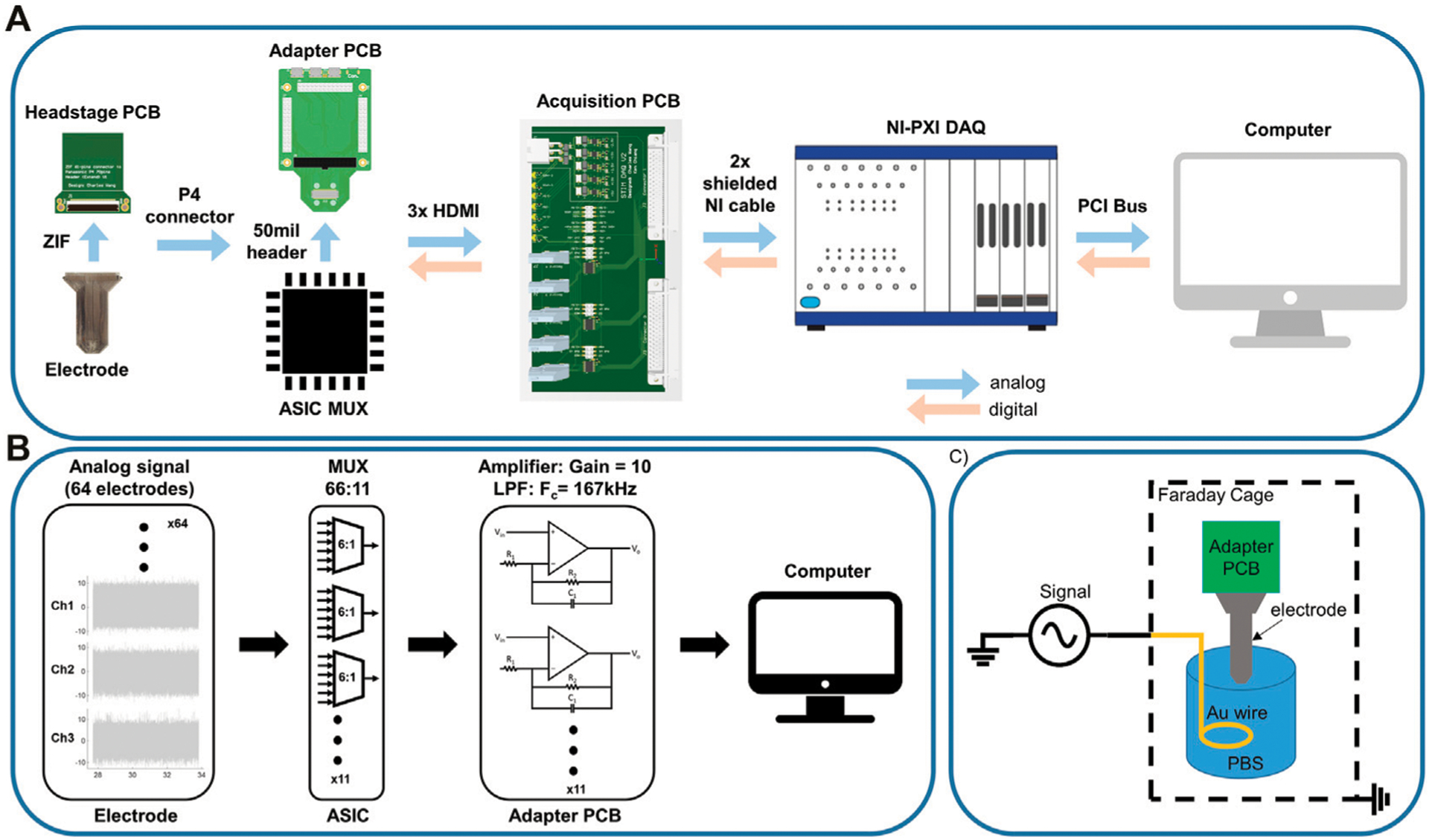
Recording setup. **(A)** Analog recording setup: Electrode array used a ZIF connector to attach to the headstage PCB. Headstage PCB connected to the adapter PCB *via* P4 connector. The adapter PCB passed signals to the ASIC MUX *via* 50mil pin connectors and relayed the signal to the acquisition PCB through 3 × μHDMI cables. The acquisition PCB passed the signal to the NI DAQ, where the ADC sampled the signal and parsed it into 61 channels at 950SPS per electrode channel. The NI also sent digital signals (clock, switching) to the ASIC MUX. **(B)** Signal processing pipe-line: Analog signals sensed at the electrodes were multiplexed on the ASIC MUX and low-pass filtered (F_c_ = 167 kHz) and amplified (Gain = 10) on the adapter PCB (non-inverting lowpass filter configuration). Filtered signals were then passed to the ADC to be parsed back into 61 channels and decimated (middle four of every 8) and averaged to avoid edge effects of switching. **(C)** Noise and signal recording setup in saline. The whole system is placed in a Faraday cage to reduce ambient noise. The electrode reference is attached to the ground of the signal generator. A gold wire is immersed saline as reference electrode.

To allow for digital and analog communication between the ML1664 ASIC and the recording system (National Instruments, NI-PXI DAQ), we developed an adapter printed circuit board (PCB) to attach between the electrode array and the acquisition system we previously developed (Insanally et al., 2016). The electrode array was attached to a headstage PCB using a ZIF connector (HIROSE Inc., FH43 series, 61 pin). The headstage PCB was attached to the adapter PCB using a board-stacking connector (Panasonic P4 series, Panasonic Corporation, Kadoma, Osaka, Japan). The adapter PCB connected to the ASIC multiplexer chip using 50 mil header connectors (Figure 3A). Eleven 6:1 multiplexers on the ASIC routed the 61 electrode connections to 11 output wires. Due to size and wiring constraints, only 11 output lines were wired out to the connector. The outputs were buffered on the adapter PCB using op-amps (Texas Instruments, Inc., OPA4140) in a non-inverting, active low-pass filter (F_c_ = 167 kHz) configuration with a gain of 10 (Figure 3B). The filtered and amplified analog signals were then passed *via* 3 μHDMI cables to an acquisition PCB, which were then connected to the DAQ (National Instruments PXI-6289) *via* shielded NI cables, where they were digitized and passed to a computer. The DAQ was controlled using LabVIEW software (LabVIEW, National Instruments) to demultiplex the signal back to 64 channels and provide digital controls to the ASIC. The sampling rate was 500,000 Samples per Second (SPS) using the NI card. To reduce the edge effects of switching, for every eight consecutive samples two edge samples on each end were truncated (first two and last two samples) and the remaining middle four samples were averaged. With 11 inputs, parsed into six electrodes per input, and the above truncation and averaging, the sampling rate per channel was ~1,000 SPS.

### *In-Vitro* Testing

The ASIC noise was measured in two configurations: 1) with the input of the multiplexers directly grounded on the headstage PCB and 2) with the electrode array attached and grounded in phosphate-buffered saline (PBS) using a gold wire (Figure 3C). Noise measurements were also completed using the same electrodes and a conventional electrophysiology system (Intan RHD2164) to compare our system to current state-of-the-art recording technology. Additionally, the electrode noise performance was compared to previously developed electrode arrays of similar form factor that were fabricated on an LCP substrate. The fabrication and performance of these electrode arrays were summarized in previous work (Woods et al., 2017; Chiang et al., 2021).

### Accelerated Aging Tests

**FIGURE 4 |:**
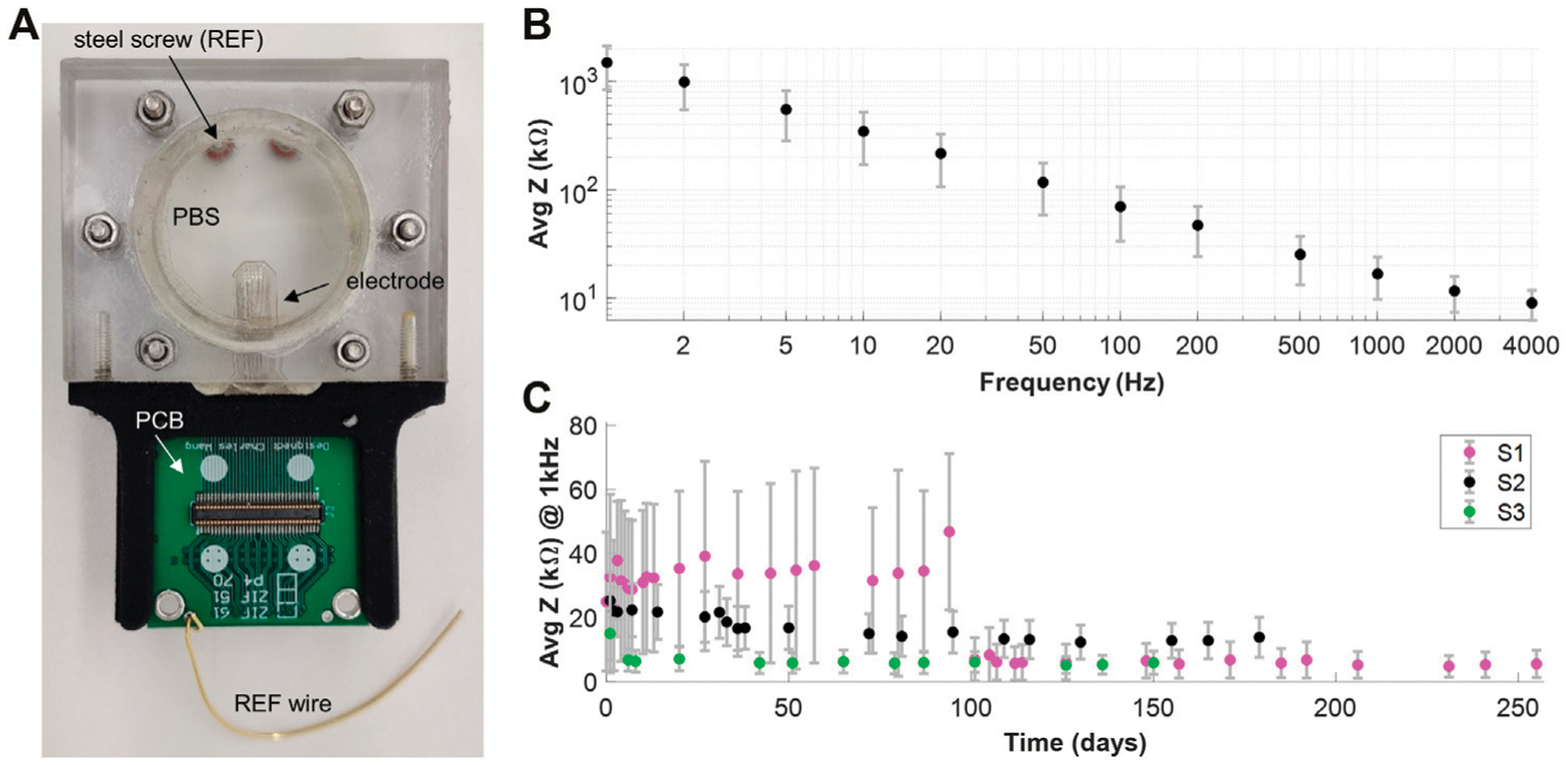
Impedance Measurements. **(A)** Soaking case setup for accelerated aging tests. An acrylic case with a circular window holds the electrode array in saline. The outside of the case is sealed with silicone (Sylgard 184) to avoid evaporation of the saline. A steel screw immersed into the saline acts as a reference electrode. The PCB allows connection of the electrode array for impedance measurements without ever extracting it from the soak case. The soak case was kept at 60°C for these recordings for accelerated aging. **(B)** EIS measurements of a monolayer silicone electrode array. **(C)** Accelerated aging tests results for 3 monolayer silicone electrode arrays.

We designed and fabricated an accelerated aging case using acrylic sheets with a hollow opening to hold the electrode array soaked in PBS at 60°C (Figure 4A). The case was sealed with silicone (Sylgard 184) to prevent saline evaporation. This provided an acceleration of approximately 5 times according to the Arrhenius equation (Hukins et al., 2008). A 3D-printed holder attached a PCB to the immersed electrode array to allow for easy connection to an impedance testing system (NanoZ Impedance Tester) while the acrylic casing protected the PCB from moisture exposure. Electrical impedance spectroscopy (1–4000 Hz) was performed to evaluate electrode survival over time (NanoZ v1.4.0). Contacts with impedance in the range of 2–500 kΩ were considered functioning channels, with anything below assumed to be shorted and above to have a broken trace.

### Surgical Procedure

Animal procedures were conducted under an approved protocol by the Duke University Institutional Animal Care and Use Committee. Female Sprague-Dawley rats weighing 225–275 g were used. Isoflurane (Patterson Veterinary) gas was delivered through a vaporizer (Absolute Anesthesia) *via* nose cone (induction 3% at 1.0 L/min and maintenance at 1–3% at 0.6 L/min) to anesthetize the animal. The head was secured using ear bars, shaved, and sterilized with alcohol and iodine wipes. Puralube was applied to the eyes to preserve moisture and reduce irritation. Subcutaneous injections of buprenorphine SR-LAB (1.2 mg/kg; ZooPharm) for pain management, dexamethasone (0.3 mg/kg) to reduce cerebral swelling, and atropine (0.02 mg/kg) to reduce salivation and bronchial secretion were performed. Lidocaine (4%) was administered topically to the site of the incision to minimize pain at the wound post-surgery. Surgery involved a longitudinal incision along the midline of the head and removal of periosteal membranes to expose the skull. Five bone screws (00–96 × 3/32; Plastics One) were drilled (Dremel 4000; 0.8 mm drill bit) into the skull on the opposite side of the hemisphere from where the craniotomy was performed to avoid damage to the brain tissue of interest. The left temporalis muscle was reflected and a 5 × 5 mm^2^ craniotomy was performed over the left auditory cortex. A sterilized electrode array attached to a connector PCB was placed epidurally over the core auditory cortex using vascular landmarks and placement was optimized by measuring impedance and testing for evoked responses to auditory stimuli across the array. The back of the array was covered by a gelatin sponge (GELFOAM, Pfizer Inc.) and secured with dental cement (C and B Metabond Quick! Luting Cement). Silver grounding wires were wrapped around the five bone screws to provide an electrical recording reference and act as anchors for the dental cement. The remaining exposed skull, electrode array and connector PCB were covered with a dental acrylic (LANG Jet Denture Repair). The incision was sutured and postoperative antibiotics were administered twice daily for 3 days post-op. Initial electrophysiological recordings were done seven days following implantation to allow the animal time to heal and for reduction of the short-term immune response at the implantation site.

### *In-Vivo* Recording

The electrode array was attached *via* ZIF connector to a head-mounted PCB that allowed for connecting to the recording system *via* the P4 connector. During the recording session the rat was placed in a sound-attenuated Faraday cage. The head-mounted PCB was attached to the female end of the P4 connector on one of the two recording systems: ML1664 ASIC system in acute studies or the conventional electrophysiology system (Intan RHD 2164 64-channel amplifier) for chronic studies and comparison recordings. Magnets on the head-mounted PCB and adapter PCB provided self-alignment for fast and easy connection on the awake and freely moving animal. A flexible SPI tether cable allowed the animal to freely move throughout the recording procedure. Data was sampled at ~1 kSPS on the ML1664 ASIC system and 20 kSPS on the conventional system. For the experimental task, acoustic stimuli were generated with custom MATLAB code through an NI 6289 DAC card and delivered through a free-field speaker (CR3, Mackie) calibrated to have a flat output over the frequency range used. Responses to “clicks”, audio stimuli of uniform power across all frequencies, were recorded to optimize placement of the electrode array to get maximal response across the array. Responses to pips of 13 frequencies (0.5–32 kHz, 0.5 octave spacing, 50 m duration, 2 m cosine-squared ramps) at 70 dB SPL were recorded for tonotopic mapping. Tones were presented in a pseudorandom sequence at a rate of ~1 Hz; each tone was repeated for 30 trials. Acute experiments were performed using the novel reinforced silicone electrode arrays and LCP electrode arrays for comparison. Additional description of these methods can be found in our previously published work by Woods et al. (Woods et al., 2017).

### *In-Vivo* Data Analysis

Data analysis was performed using established methods in our lab that are described in detail in Woods et al. (Woods et al., 2017). Briefly, data for each channel was bandpass filtered (2–200 Hz) to isolate the local field potential (LFP). The trigger of the stimulus was used to window responses following each auditory tone played (5–80 m). Channels with more than 5% outliers were removed from the classifier. Detectability of evoked responses was measured using evoked signal-to-noise ratio (ESNR) by evaluating the ratio of response to baseline RMS. For tone-evoked SNR, the maximum-response tone frequency was used. Tone decoding accuracy and average error of classification were then determined by measuring the octave difference between the predicted and true tone.

## RESULTS

### ASIC Performance

The ASIC consumed 20 μA when operating from 10 kHz ±5 V AC power. The digital control of the multiplexer was provided using a differential, balanced data signal with Manchester encoding. The multiplexer was clocked from the AC power line and the switching rate of the multiplexer was proportional to the clock. The AC power could be driven from 10–500 kHz to enable a faster or slower switching speed for changing between multiplexer registers. The multiplexer had several independent registers which could be pre-loaded to connect a pre-defined montage of electrodes. Then on each clock cycle, the multiplexer would rapidly switch between pre-configured registers to enable electrode rastering to sample a large-scale of electrodes across a wide area. The multiplexer may also be dynamically controlled, subject to the data rate of programming the multiplexer at ~1 kHz. Each register had a corresponding electrode output cell which had the ability to be selected as high impedance, connected, or grounded (Figure 2A).

### Noise Measurements

Bench-top testing of the recording system was performed to characterize the quality of the acquired signal through the custom ASIC and the connecting hardware. Performance was compared to identical noise recordings taken using a conventional neural recording system (Intan Technologies). Noise was calculated by recording signal as described in Figure 2C and calculating the average root mean squared of data from all functional electrode contacts filtered between 2–200 Hz using a 6th order digital Butterworth filter. With inputs shorted to reference, the ML 1664 ASIC had 2.65 μV rms noise, as compared to 1.11 μV rms on the Intan recording system. After attachment of the silicone electrode array, the noise measured 3.03 μV rms and 2.50 μV rms for the MUX and Intan respectively. We additionally tested the noise with a gold LCP electrode array that had slightly larger impedance (Supplementary Figure S1), which yielded 5.33 μV rms and 3.67 μV rms for the MUX and Intan respectively. In addition to noise testing, we tested the cross talk between channels of our recording system by calculating cross-correlation between all channels. The cross correlation was lowest when the inputs to the ASIC were shorted to the reference. The cross-correlation was also dependent on the impedance of the electrode attached to the system, with higher impedance electrodes showing greater cross-correlation than lower impedance electrodes (Supplementary Figure S1).

### Impedance Measurements

EIS measurements of the electrode array revealed impedance of 17 +− 3 kΩ at 1 kHz and a linear trend of decreasing impedance when graphed *vs*. increasing frequency on a log-log scale (Figure 4B). Accelerated aging tests were conducted on three monolayer silicone electrode arrays over the course of over 100 days to test for delamination and longevity of the device. The electrode arrays showed consistently low impedance throughout the duration of soaking and had consistent yields (80–90%), indicating no conductor wire breakage. Cross-correlation tests were performed on the aging tests to ensure that the electrode arrays were not delaminating and no water ingress has occurred. The cross-correlation did not differ between fresh samples and samples soaked, suggesting no substantial water ingress that would short the contacts together.

### Acute *In-Vivo* Data

**FIGURE 5 |:**
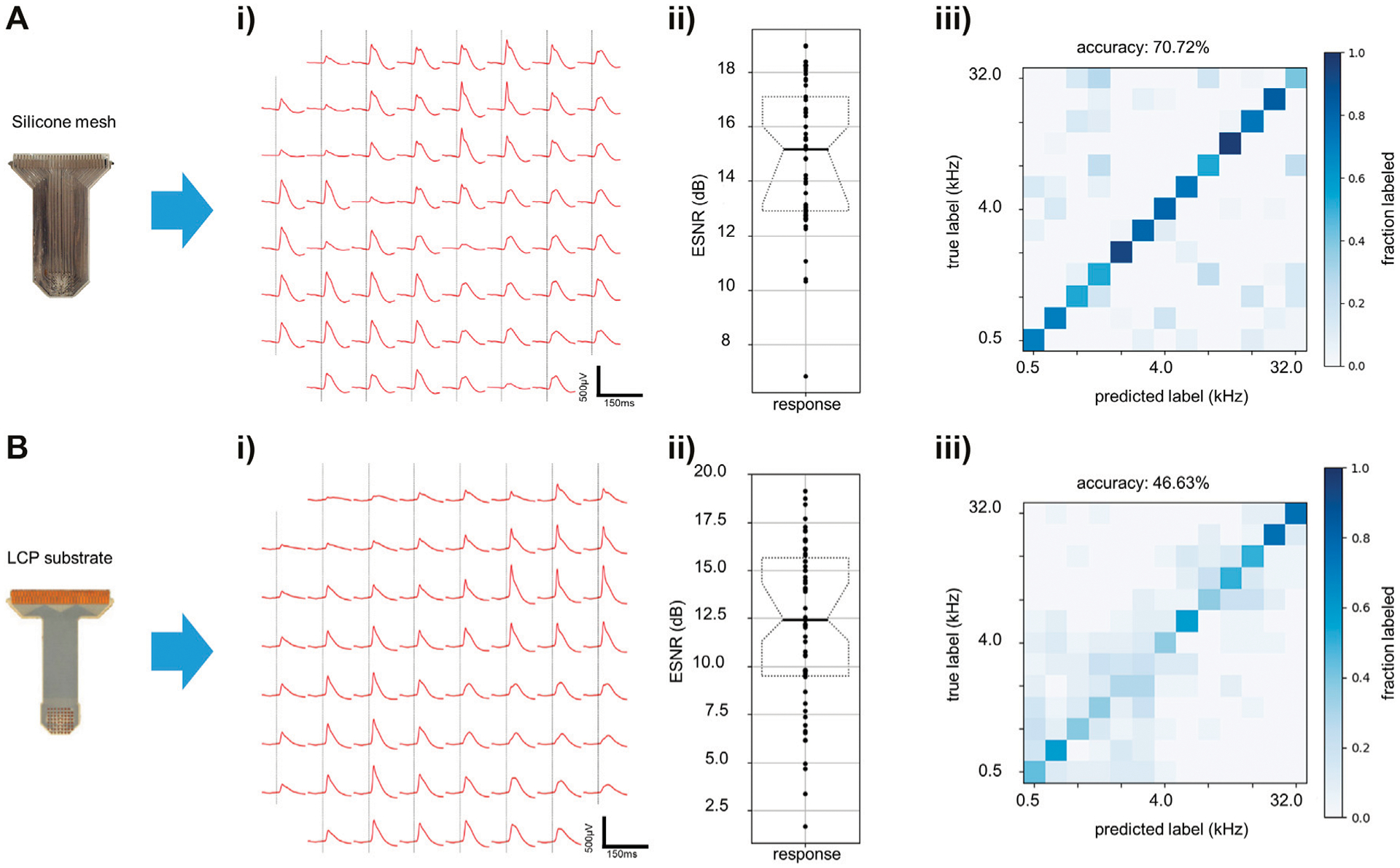
Electrode recording capability testing. **(A)** Silicone electrode *in vivo* results. **(i)** Evoked responses to auditory clicks using reinforced silicone electrode array recorded using a conventional recording system (Intan). **(ii)** ESNR for “click” stimulus on all contacts on reinforced silicone electrode array. **(iii)** Prediction accuracy of recordings using reinforced silicone electrode array with 13 auditory stimulus tones (chance = 8%). **(B)** LCP electrode array *in vivo* results. **(i)** Evoked responses to clicks for LCP electrode array recorded on Intan. **(ii)** ESNR for “click” stimulus on all contacts on LCP electrode array. **(iii)** Prediction accuracy of recordings using LCP electrode array for 13 stimulus tones (chance = 8%).

**FIGURE 6 |:**
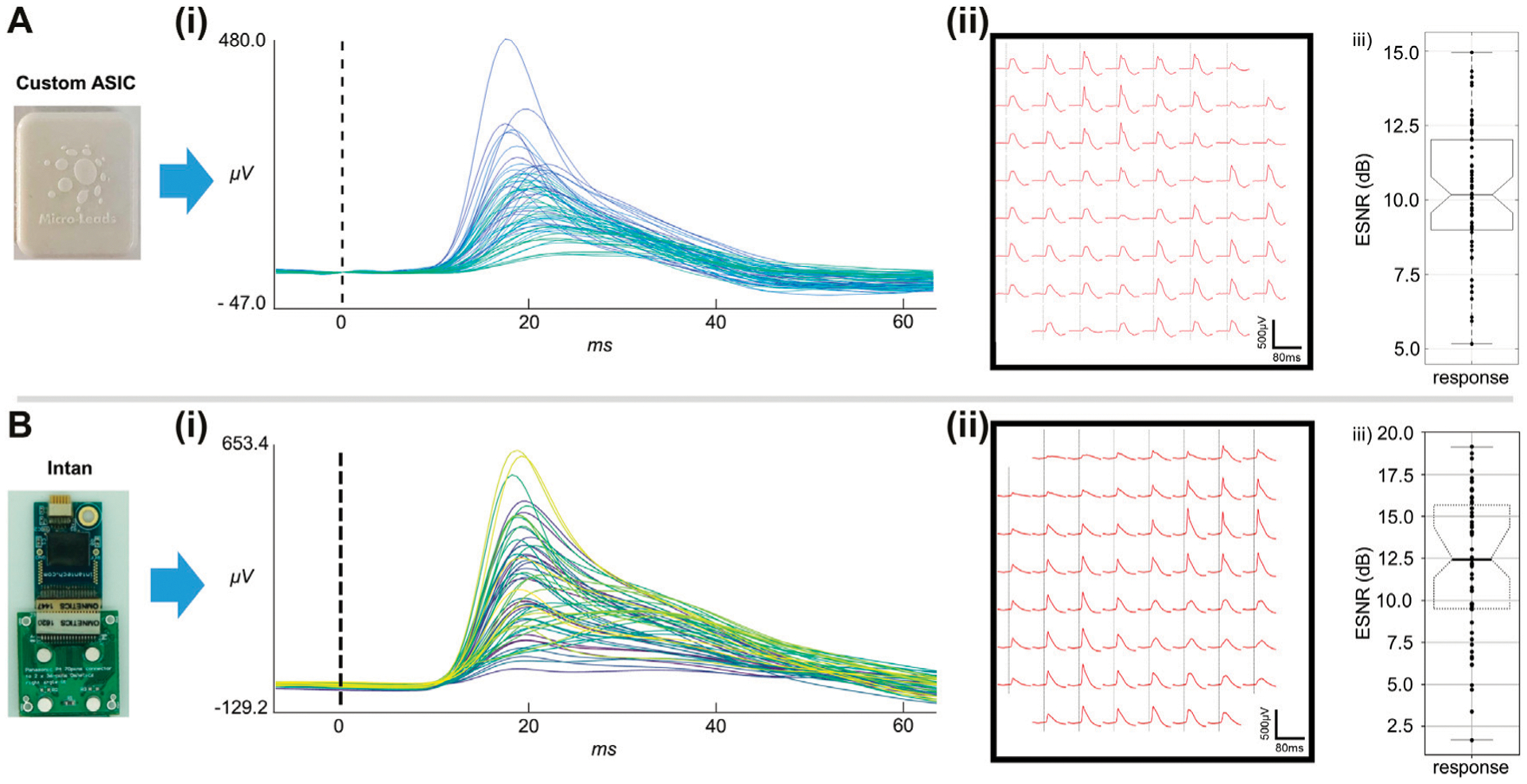
Acute *in-vivo* recordings. **(A)** *In-vivo* recordings using an LCP-substrate electrode connected to the custom ASIC MUX. **(i)** Averaged evoked responses from all 61 electrode channels. **(ii)** Averaged evoked potentials at each electrode site on the 61 channel array. **(iii)** ESNR for the ASIC MUX recordings. **(B)** *In-vivo* recordings using an LCP-substrate electrode connected to conventional recording system (Intan). **(i)** Averaged evoked responses from all 61 electrode channels. **(ii)** Averaged evoked potentials at each electrode site on the 61 channel array. **(iii)** ESNR for the Intan headstage recordings.

We compared the performance of a monolayer passive reinforced silicone electrode array to that of an LCP electrode array on the Intan recording system. The electrode arrays showed similar performance in terms of averaged evoked response peak-to-peak voltage (Figures 5Ai,Bi), ESNR (Figures 5Aii,Bii), and stimulus tone decoding (Figures 5Aiii,Biii), with the reinforced silicone electrode array slightly outperforming the LCP electrode array (Figure 5). We next compared the recording ability of the ML1664 ASIC to the Intan headstage using the same LCP electrode array. Acute recordings form the auditory cortex of the rat showed strong evoked responses to sound stimuli up to 520 μV_pp_ using the custom ASIC (Figure 6Ai). The signals had comparable magnitudes using the Intan headstage up to 780 μV_pp_ (Figure 6Bi). ESNR was high for both the custom ASIC and the Intan headstage: 10 and 12.5 dB respectively, demonstrating the ability to multiplex and record distinct brain signals from densely spaced electrode contacts on the cortex with a high signal-to-noise ratio (Figures 6Aiii,Biii).

### Chronic *In-Vivo* Data

**FIGURE 7 |:**
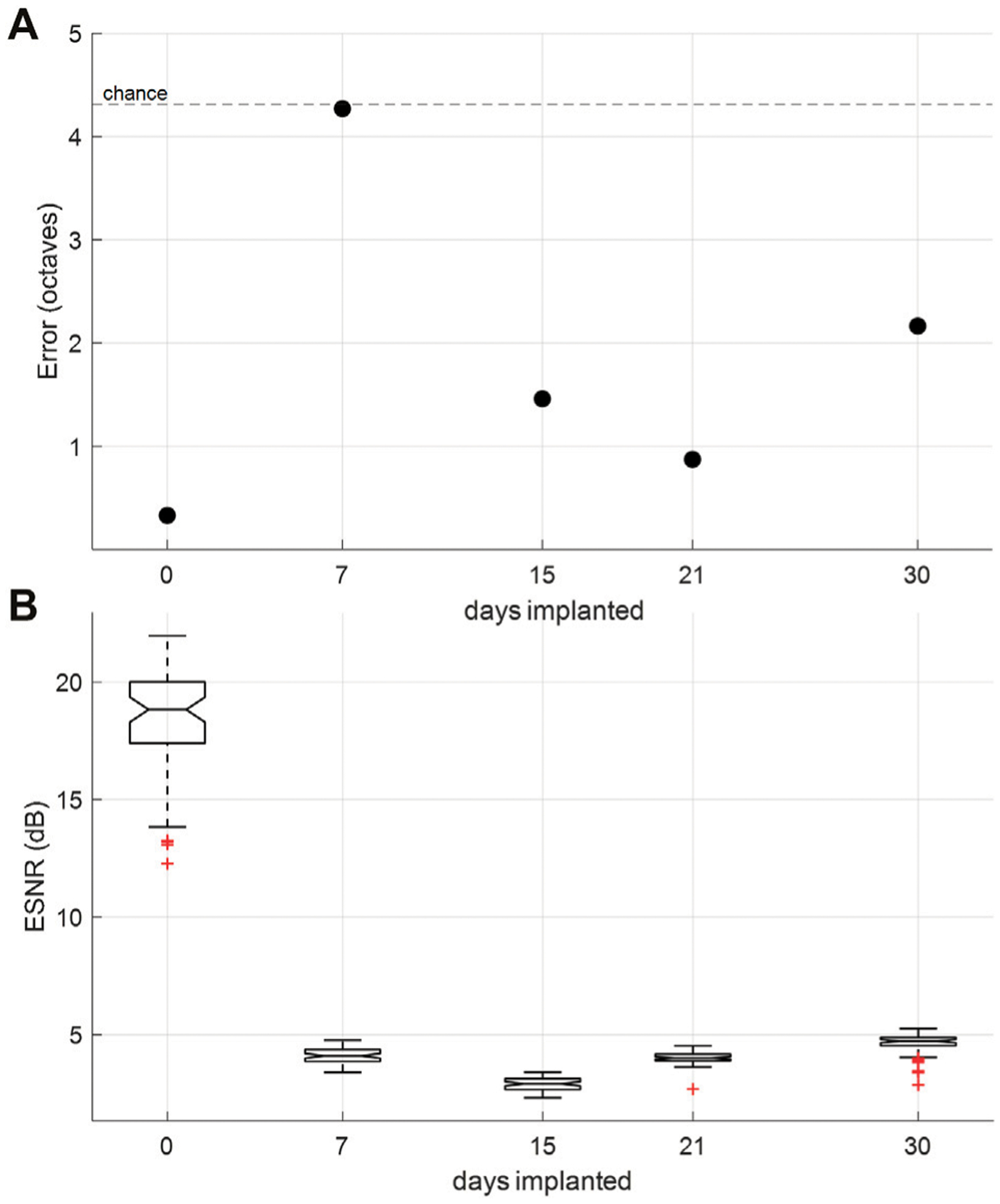
Chronic *in-vivo* recordings. **(A)** Tone-decoding error over time. Decoding 13 tones played (0.5–32 kHz) at half-octave spacing, which provides chance at 4.31 octaves. **(B)** ESNR over time measured by ratio of SNR during evoked potential to SNR during idle behavior (200 m of recording prior to auditory stimulus).

A monolayer reinforced silicone electrode array was implanted chronically over the auditory cortex of the rat using method described in 2.8. An intraoperative recording was conducted before sealing the craniotomy and scalp to ensure good electrode array placement and showed strong ESNR: ~20dB (Figure 7A, day 0) and decoding accuracy: 73% (Figure 7B, day 0). The rat was given seven days to recover after the implantation and then underwent weekly recordings over a period of 30 days. ESNR to “click” stimuli and decoding error of the 13 tones played initially worsened significantly after seven days of implantation, but subsequently recovered, showing ESNR in the range of ~5 and decoding error well below chance for the last three weeks of implantation (Figures 7A,B).

## DISCUSSION

In this work, we tested the capability of fabricating a neural interface from novel materials and attempt to combine flexible substrates with standard silicon electronics to yield a high-channel implantable device with few output wires. We report fabrication methods with 50 μm pitch and trace on silicone substrate using laser ablation to fabricate high-density μECoG arrays. The fine metal patterning also allowed for placement of a footprint to attach a custom designed ASIC MUX *via* pin-grid array and compression thermobonding. We separately tested the reinforced silicone electrode array performance and the ASIC MUX performance and compared both *in vitro* and *in vivo* to conventional electrode arrays and recording systems in our lab: LCP electrode arrays and an Intan headstage respectively. Overall, we demonstrated durability of the reinforced silicone electrode array through *in vitro* accelerated aging tests and recording ability through *in vivo* recordings over the rat auditory cortex. The custom-designed ML1664 ASIC was compared to a standard recording system (Intan) and showed ability to record with low noise and sense evoked-responses with spatial variance across the auditory cortex that allow for tonotopic mapping while providing an almost four-fold reduction in wire count from 61 to 16 wires. In future work, the multiplexing ratio could be increased to further scale the device and more advanced circuitry could be incorporated for on-site amplification, AD conversion or wireless capability to improve device performance.

Fabrication techniques presented in this work demonstrated the ability to pattern at 50 μm trace and space on a reinforced silicone substrate, enabling high-density contacts for the electrode array. Using laser ablation on conductive foil was advantageous as it allowed for more precise and finer patterning than current methodologies (CorTec, 2021). Compared to other electrode substrates like Polyimide, MED-4174 silicone has a low Young’s modulus of 8.28 MPa which minimizes damage to interfacing tissue. Without reinforcement in the silicone, the platinum-iridium conductors are prone to breakage due to the uneven distribution of applied stress between the silicone and the platinum-iridium when under an applied load. The addition of PET reinforcement fibers in the silicone redistributes the applied stress more evenly and improves the reliability of the platinum-iridium traces. Additionally, the reinforced silicone allowed for easy handling of the electrode array as the risk of tearing the substrate was decreased due to the embedded fibers. Fabricating the monolayer electrode array from Pt-Ir foil provided sufficiently low impedance for recording. To further improve impedance, we could roughen the exposed platinum-iridium, or electroplate the contacts with a platinum-iridium coating to increase the effective surface area. Additionally, since the opening to the contacts was ablated into a 100 μm thick silicone layer, it took additional time for the contacts to become fully “wetted”, as moisture needed to fill the well over the electrode to establish contact with the saline. This can be seen in the decrease of impedance in sample S1 after some duration of being immersed in saline in accelerated aging tests (Figure 4C). By jetting the sites with a syringe prior to immersion or modifying the design and raising the metal to the surface of the silicone superstrate the wetting procedure and therefore the impedance could be improved. The wetting of the well above the electrode posed issues for the deeper contacts on our two-layer silicone electrode arrays. With limited contact to saline or neural tissue it was not possible to acquire *in vitro* or *in vivo* results for the two-layer devices. The above-mentioned solution of raising the metal or decreasing thickness of the superstrate could be applied in future work to improve the interface to the second-layer electrode contacts.

The ML1664 ASIC showed sufficiently low noise and recording capability, with noise and correlation between channels being best when using low impedance electrodes. In this work, the electrode array and MUX were tested separately, so additional testing must be done to determine performance of the MUX attached to the reinforced silicone electrode array. Additionally, the survival of the LCP-encapsulated ASIC chip in saline and *in vivo* must still be determined to characterize the longevity of this device design.

Acute *in vivo* recordings using the ASIC MUX comparing the reinforced silicone electrode arrays showed improved ESNR and decoding as compared to the LCP electrode arrays, despite having higher impedance. This may be due to the greater conformability and softness of the reinforced silicone electrode array, allowing for more proximate measurements from the cortex and thereby improved SNR and spatial sampling. We also cannot exclude the possibility that imprecise placement on the auditory cortex allowed for better data acquisition with one electrode array over the other. Precise placement was challenging due to size discrepancy between the two electrodes arrays with the silicone electrode array having wider margins than the LCP electrode array and the limits of exposed auditory cortex through the craniotomy.

Chronic *in vivo* testing of the reinforced silicone electrode array showed recording ability of the electrode array over 30 days. A dramatic decrease in ESNR and decoding accuracy from the intra-operative recordings to subsequent implanted recordings may suggest that the acute immune response worsened the interface between the electrode array and brain surface post implantation. This was accentuated on day 7, when ESNR is at its lowest and decoding error rises to chance, but as the immune response decreases the recording quality improved in the subsequent weeks. Additionally, it is important to note that intra-operative recordings were done with the animal under anesthesia, while subsequent recordings are done with the animal awake and free roaming. Awake recordings have lower ESNR as baseline brain activity is no longer suppressed by the anesthesia. This has been observed in our previous animal recordings (Woods et al., 2017). Overall, the ESNR and decoding quality for the silicone electrode array were comparable to chronic recordings using our LCP electrode arrays, demonstrating functional survival of the reinforced silicone electrode array *in vivo* over a period up to at least 30 days. Although the electrode was still functional on day 30, we were forced to halt the chronic recording due to an unrelated health condition.

## CONCLUSION

In this work, we present fabrication methods for a high-resolution active electrode array on a novel reinforced silicone-mesh substrate and an attached, on-site multiplexing chip for high-resolution sampling of electrical data from the cortex. The novel active electrode array demonstrated the ability to sense intricate electrophysiological data over a large number of electrodes above the rat auditory cortex for up to 30 days using active multiplexing. The ASIC multiplexing chip, near-hermetic package, and methods of attachment between the electronics package and the substrate are also presented. Functionality of the ASIC MUX was compared to a conventional recording system. These methodologies provide options for developing highly conformal, spatially dense electrode arrays, as on-site multiplexing allows for scaling of electrode contact count while minimizing output wires and thereby reducing the footprint of the device at the implantation site. We expect this work to enable the development of novel, high-resolution, high-channel count devices that allow for improved interfacing with the nervous system.

## Supplementary Material

supp **Supplementary Figure S1 |** Correlation for MUX channels at different impedances. **(i)** Grounded inputs. Left: Correlation coefficients matrix. Right: Average correlation rank for each channel (11 output columns, 6 channel rows per output). **(ii)** *Z* = 3.4 kΩ (Pt-Ir coated contacts, LCP electrode array). Left: Correlation coefficients matrix. Right: Average correlation rank for each channel (11 output columns, 6 channel rows per output). **(iii)** *Z* = 27.0 kΩ (Pt-Ir foil, silicone electrode array). Left: Correlation coefficients matrix. Right: Average correlation rank for each channel (11 output columns, 6 channel rows per output). **(iv)** *Z* = 38.5 kΩ (Au contacts, LCP electrode array). Left: Correlation coefficients matrix. Right: Average correlation rank for each channel (11 output columns, 6 channel rows per output).

## Data Availability

The original contributions presented in the study are included in the article/Supplementary Material, further inquiries can be directed to the corresponding author.
