# Supplementary material for: High-Density, Actively Multiplexed μECoG Array on Reinforced Silicone Substrate
Source: Front Nanotechnol. Author manuscript; Available in PMC 2022 Nov 24. (PMC9310058; doi:10.3389/fnano.2022.837328)
Supplement: supp — lementary Figure S1 | Correlation for MUX channels at different impedances. (i) Grounded inputs. Left: Correlation coefficients matrix. Right: Average correlation rank for each channel (11 output columns, 6 channel rows per output). (ii) Z = 3.4 kΩ (Pt-Ir coated contacts, LCP electrode array). Left: Correlation coefficients matrix. Right: Average correlation rank for each channel (11 output columns, 6 channel rows per output). (iii) Z = 27.0 kΩ (Pt-Ir foil, silicone electrode array). Left: Correlation coefficients matrix. Right: Average correlation rank for each channel (11 output columns, 6 channel rows per output). (iv) Z = 38.5 kΩ (Au contacts, LCP electrode array). Left: Correlation coefficients matrix. Right: Average correlation rank for each channel (11 output columns, 6 channel rows per output). [file NIHMS1818773-supplement-supp.pdf]

Supplementary Material:

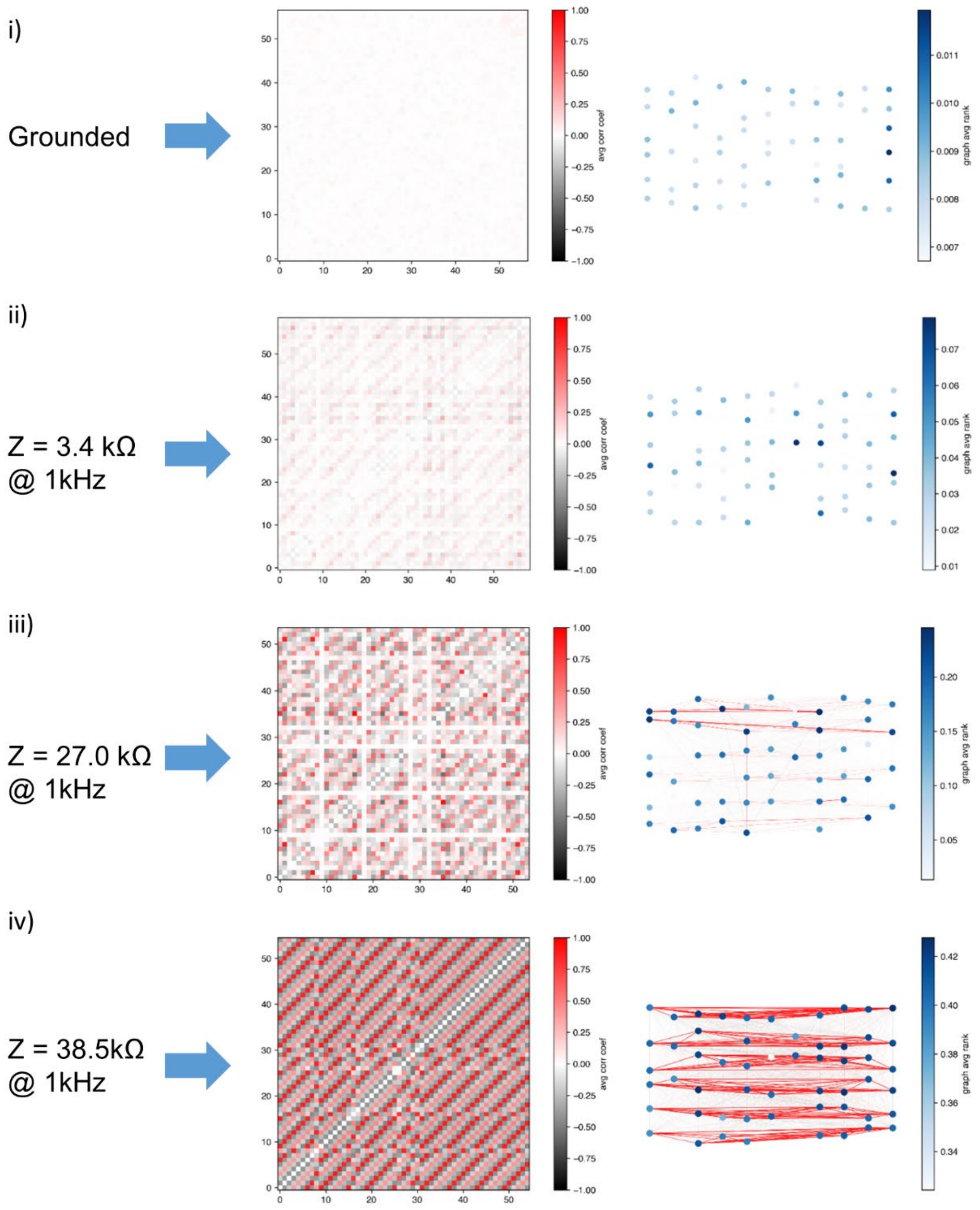

Figure S1: Correlation for MUX channels at different impedances. i) Grounded inputs. Left: Correlation coefficients matrix. Right: Average correlation rank for each channel (11 output columns, 6 channel rows per output). ii) Z = 3.4 kΩ (Pt-Ir coated contacts, LCP electrode array). Left: Correlation coefficients matrix. Right: Average correlation rank for each channel (11 output columns, 6 channel rows per output). iii) Z = 27.0 kΩ (Pt-Ir foil, silicone electrode array). Left: Correlation coefficients matrix. Right: Average correlation rank for each channel (11 output columns, 6 channel rows per output). iv) Z = 38.5 kΩ (Au contacts, LCP electrode array). Left: Correlation coefficients matrix. Right: Average correlation rank for each channel (11 output columns, 6 channel rows per output).
